# 간간호대학생의 우울, 피로, 신체존중감은 월경전 증상에 영향을 미치는가?

**DOI:** 10.4069/kjwhn.2020.09.10

**Published:** 2020-09-29

**Authors:** Eun Joo Lee, Seung Kyoung Yang

**Affiliations:** Department of Nursing, Kyungnam University, Changwon, Korea; 경남대학교 간호학과

**Keywords:** Body image, Depression, Fatigue, Premenstrual syndrome, Nursing students, 신체상, 우울, 피로, 월경전증후군, 간호대학생

## Abstract

**Purpose:**

The purpose of this study was to investigate factors affecting premenstrual symptoms among nursing students, focusing on depression, fatigue, and body esteem.

**Methods:**

The participants were 145 nursing students at a university located in Changwon, Korea. Data were collected from November 2 to November 30, 2019 using self-reported structured questionnaires, and analyzed using descriptive statistics, independent t-test, analysis of variance, Pearson correlation coefficients, and multiple regression analysis.

**Results:**

The mean item score for premenstrual symptoms was 2.52±0.92, indicating a low level. The mean score for depression was 16.05±7.72, and 15.2% of participants were found to be moderately depressed and 9.7% severely depressed. The mean item score for fatigue was 4.84±0.84, indicating a moderate level, and body esteem was 2.94±0.44, indicating a moderate level. The premenstrual symptoms of nursing students showed a statistically significant correlation with depression (r=–.58, *p*<.001), fatigue (r=.33, *p*<.001), and body esteem (r=–.28, *p*<.001). Factors impacting premenstrual symptoms of nursing students were depression (β=.47, *p*<.001), dysmenorrhea (β=–.18, *p*=.009), menstrual cycle irregularity (β=.17, *p*=.013), and body esteem (β=–.14, *p*=.038). The total explanatory power of these variables was 41.0%.

**Conclusion:**

Findings from this sample of nursing students suggest that intervention programs to relieve premenstrual symptoms should focus on depression, menstrual cycle regularity, dysmenorrhea, and body esteem.

## Introduction

### 연구의 필요성

월경전증후군이란 월경이 시작되기 전 일상생활에 지장을 줄 정도의 다양한 신체적, 정서적, 행동적 불편감을 경험하게 되는 것을 의미한다[1]. 월경 전 증상은 매우 다양하며 유방압통, 사지부종, 복통, 두통, 체중 증가, 복부 팽만감 등의 신체적 증상과 짜증, 불안, 긴장, 우울, 공격성 등의 정서적 증상 외에도 피로, 수면 장애, 흥미 저하, 사회적 위축, 주의 집중 곤란 등의 행동적 증상이 나타나기도 한다[2]. 월경전증후군의 유병률은 측정 방법과 대상에 따라 차이가 있으나 가임기 여성의 20%–40%는 일상생활에 지장을 받을 정도로 심한 월경전증후군을 경험하는 것으로 알려져 있다[2].

선행연구에 따르면 우리나라 여대생의 98%까지 월경 전 증상을 경험하고 있는 것으로 보고되었으며[3], 연령대별로 나누어 살펴보았을 때 대학생 시기에 해당하는 20대의 경우 중년과 고등학생 시기에 비해 월경 전 증상이 심한 것으로 나타났다[4]. 하지만 월경전증후군에 대한 인지도가 낮고, 미혼 여성의 산부인과 진료에 대한 사회적 편견으로 인해 대부분의 경우 적극적인 치료보다는 스스로 감내하려는 소극적이거나 회피적인 방법을 선택하고 있다[5]. 이러한 월경전증후군은 여성의 삶의 질 저하의 원인이 되며[6], 특히 월경전증후군의 유병률이 높은 시기에 속하는 여대생은 이로 인해 스트레스, 학업 능력 저하, 부정 행위, 범죄나 자살까지 초래할 수 있으므로[3] 여대생을 대상으로 적극적인 월경 전 증상 관리가 필요하다.

월경전증후군의 명확한 원인은 아직까지 밝혀지지 않은 상태지만, 호르몬 불균형, 신경 전달 물질 변화 등의 생물학적 요인과[7], 우울[8], 월경에 대한 태도, 스트레스[9] 등과 같은 심리·사회적 요인들 간의 상호작용과 관련 있는 것으로 보고되고 있다. 이러한 월경전증후군은 심리·사회적 요인의 영향을 크게 받는 것으로 알려져 있다[9].

대학생은 청소년기를 벗어나 대학 생활이라는 새로운 환경적 변화로 인해 혼란과 갈등을 경험하게 되며, 특히 간호대학생은 과중한 학업 부담감과 국가고시, 임상실습 등에 대한 심리적 압박감으로 인해 스트레스와 우울 같은 정신적 문제를 호소하는 것으로 나타났다[10]. 이러한 우울은 월경전증후군과 관련 있는 것으로 보고되고 있으며[8], 간호대학생의 경우 타 학과 학생들에 비해 우울 정도가 높은 것으로 나타났으므로[11], 간호대학생을 대상으로 월경 전 증상 정도를 살펴보는 것은 의미 있는 일이라 생각된다.

피로감은 월경 전 증상 중 하나로 보고되고 있으나, 아직까지 월경전증후군에서 어떠한 기전으로 피로가 발생하는지에 대한 연구는 불충분한 수준이다[2]. 일상생활에서 간호대학생은 이론수업과 임상실습을 병행하며 타 학과 학생보다 더 많은 피로감을 호소한다고 하였는데[12], 간호대학생의 피로 유발 요인으로는 수면 부족, 과제, 임상실습 스트레스 등으로 나타났다[13]. 피로는 우울과도 관련 있으며[12], 월경 전 신체적 증상과 맞물러 가중될 수 있으므로 적절한 피로 관리가 필요할 것으로 생각된다.

신체존중감이란 자기존중감의 구성요소로 자신의 신체나 외모에 대한 자기 평가를 의미하며, 신체를 다루는 방식 및 사회적 관계 행위와 관련 있다[14]. 여성의 경우 외모와 체중에 근거하여 신체존중감과 자아존중감이 형성되기도 한다[14]. 이러한 신체존중감은 삶의 질, 대인관계, 건강증진 행위 등에 영향을 미치게 되며, 낮은 신체존중감은 스트레스 유발로 이어질 수 있다[15]. 연구에 따르면 월경 전 증상이 심할수록 부정적인 신체상(body image)을 보이는 것으로 나타났으며[16], 신체에 대한 부정적 인식은 신체존중감 저하와 함께 스트레스, 우울 등과 같은 심리적 문제를 일으켜 월경 전 증상에 영향을 줄 수 있을 것으로 여겨진다. 여대생은 자신의 외모와 체중에 관심이 높은 시기에 속하며, 간호대학생은 성인에 비해 신체존중감이 낮은 것으로 보고되므로[17], 신체존중감이 월경 전 증상과 어떠한 관련성이 있는지 확인해 볼 필요가 있다.

지금까지 월경전증후군에 관한 연구는 건강증진 행위, 스트레스, 월경 태도, 건강 관련 생활습관 등 다양한 측면에서 이루어져 왔으며, 스트레스는 월경전증후군과 높은 관련성이 있는 것으로 보고되고 있다[9,18]. 그러나 스트레스와 관련 있는 피로, 우울, 신체존중감과 월경 전 증상과의 관계를 살펴본 연구는 찾아보기 힘들었다. 이에 본 연구에서는 간호대학생을 대상으로 피로, 우울, 신체존중감과 월경 전 증상의 관련성을 확인하고 이를 근거로 월경 전 증상을 완화시킬 수 있는 중재 프로그램의 기초자료를 마련하고자 한다.

### 연구 목적

본 연구는 간호대학생의 우울, 피로, 신체존중감이 월경 전 증상에 미치는 영향을 규명하기 위해 수행하였으며 구체적인 목적은 다음과 같다.

1) 대상자의 우울, 피로, 신체존중감, 월경 전 증상 정도를 파악한다.

2) 대상자의 일반적 특성 및 월경 관련 특성에 따른 월경 전 증상 차이를 파악한다.

3) 대상자의 우울, 피로, 신체존중감, 월경 전 증상의 상관관계를 파악한다.

4) 대상자의 월경 전 증상에 영향을 미치는 요인을 규명한다.

## Methods

Ethics statement: This study was approved by the Institutional Review Board of Kyungnam University (1040460-A-2019-014). Informed consent was obtained from the participants.

### 연구 설계

본 연구는 간호대학생의 월경 전 증상에 영향을 미치는 요인을 규명하기 위한 상관성 조사 연구설계이다.

### 연구 대상

본 연구의 대상자는 창원에 소재한 한 대학의 간호학과에 재학 중인 여대생 중 연구 목적을 이해하고 본 연구에 참여하고자 서면 동의한 자를 대상으로 하였다. G-power ver. 3.1.9를 이용하여 선행연구[18]에서 사용된 다중 회귀분석을 위한 중간 효과크기(d) .15, 검증력(1–β) .80, 유의수준(α) .05, 예측요인 14개(연령, 평균 수면 시간, 음주 빈도, 흡연, 체질량지수, 초경 연령, 월경 주기, 평균 월경 기간, 월경량, 월경통, 가족력, 우울, 피로, 신체존중감)로 하여 구하였을 때, 총 135명이 필요하였다. 불성실 답변이나 중도 탈락률 10%를 고려하여 150명에게 자료 수집을 하였으며, 그 중 응답에 불성실한 설문지 5부를 제외한 145부를 본 연구의 최종 분석에 사용하였다.

### 연구 도구

#### 월경 전 증상

월경 전 증상은 Moos [19]가 개발한 Menstrual Distress Questionnaire를 Kim과 Kwon [20]이 수정, 보완한 도구로 측정하였으며, Mind Garden (https://www.mindgarden.com/)에서 도구 구입 후 Kim과 Kwon [20]에게 사용 승인을 받아 사용하였다. 총 37문항으로 지난 월경 전 증상에 대한 자가보고 설문지이며 구성은 다음과 같다. 부정적 정서 8문항, 통증 6문항, 집중 관련 증상 8문항, 자율신경 반응 6문항, 수분 관련 증상 4문항, 행동 변화 5문항으로 증상이 ‘전혀 없다’ 1점에서 ‘아주 심한 증상이 있다’ 6점까지의 6점 척도이다. 점수 범위는 37점에서 222점까지로 점수가 높을수록 월경 전 증상 정도가 심한 것을 의미한다. 도구 개발 당시 Cronbach’s α는 .95였으며, 본 연구에서는 .96이었다. Kim과 Kwon [20]의 연구에서 하위 요인의 Cronbach’s α는 부정적 정서 .88, 통증 .86, 집중 관련 증상 .88, 자율신경 반응 .81, 수분 관련 증상 .70, 행동 변화 .61이었으며, 본 연구에서의 Cronbach’s α는 부정적 정서 .94, 통증 .87, 집중 관련 증상 .93, 자율신경 반응 .76, 수분 관련 증상 .63, 행동 변화 .87로 나타났다.

#### 우울

우울은 Radloff [21]의 자가보고형 우울 척도를 Cho와 Kim [22]이 번역한 한국판 역학연구센터 우울 척도(Center for Epidemiologic Studies Depression Scale)로 측정하였으며, Cho와 Kim [23]에게 도구 사용 승인을 받아 사용하였다. 총 20문항으로 각 문항은 ‘극히 드물다’ 0점부터 ‘대부분 그랬다’ 3점까지 4점 척도이다. 점수 범위는 0점에서 60점까지이며 점수가 높을수록 우울 정도가 높음을 의미한다. Cho와 Kim [22]의 연구에서 Cronbach’s α는 .92였으며 본 연구에서는 .83이었다. 점수에 따른 우울 정도는 0–15점은 정상, 16–20점은 경미한 우울, 21–24점은 중한 우울, 25–60점은 심한 우울로 분류하였다[23].

#### 피로

피로는 광범위한 상황과 집단의 피로를 측정하기 위해 Schwartz 등[24]이 개발한 피로 사정 도구(Fatigue Assessment Instrument)를 Byeon과 Lee [25]가 번안한 도구로 측정하였으며 도구 사용 승인을 받아 사용하였다. 총 27문항으로 각 문항은 ‘전혀 그렇지 않다’ 1점에서 ‘매우 그렇다’ 7점까지 7점 척도이며, 점수 범위는 27점에서 189점까지로 점수가 높을수록 피로가 심한 것을 의미한다. 도구 개발 당시 Cronbach’s α는 .92였으며, 본 연구에서는 .93이었다.

#### 신체존중감

신체존중감은 Gim [14]이 Gim과 Cha [26]의 문항을 기반으로 일부 문장을 수정하고 사회적 요소가 반영된 문장을 추가하여 개발한 한국판 전반적 신체존중감 척도(Korean Overall Body Esteem Scale)로 측정하였다. 도구 사용 승인을 받은 후 사용하였으며, 총 18문항으로 구성되었다. 신체기능존중감 9문항, 외모존중감 6문항, 체중존중감 3문항으로 3가지 하위 요인으로 구성되며 각 문항은 ‘전혀 그렇지 않다’ 1점에서 ‘매우 그렇다’ 4점까지 4점 척도로, 점수 범위는 18점에서 72점까지로 점수가 높을수록 신체존중감이 높음을 의미한다. Gim [14]의 연구에서 Cronbach’s α는 체기능존중감 .91, 외모존중감 .87, 체중존중감 .82이었으며, 본 연구에서의 Cronbach’s α는 체기능존중감 .86, 외모존중감 .79, 체중존중감 .71이었다.

#### 일반적 특성 및 월경 관련 특성

일반적 특성 문항은 ‘연령, 평균 수면 시간, 음주 빈도, 흡연, 체질량지수’의 5문항이었으며, 월경 관련 특성 문항은 ‘초경 연령, 월경 주기, 평균 월경 기간, 월경량, 월경통 유무, 가족력’의 6문항으로 총 11문항으로 구성되었다.

### 자료 수집

본 연구의 자료 수집은 2019년 11월 2일부터 11월 30일까지 시행하였다. 조사에 앞서 간호대학생 10명을 대상으로 예비조사를 시행하여 설문지 내용에 대한 이해도를 파악하였으며, 설문지 내용을 이해하는 데 어려움을 없음을 확인하였다. 연구 대상자가 간호대학생이므로 연구자가 직접 연구 참여자를 모집할 경우 자발적인 연구 참여가 이루어지지 않을 수 있어 연구 보조원을 선정하여 연구에 대한 구체적인 사항에 대해 교육을 실시하였다. 창원시 지역의 한 대학 간호학과에 재학 중인 1학년에서부터 4학년까지의 여대생을 대상으로 하였으며, 연구 보조원이 잠재적 연구 대상자의 수업이 없는 시간을 활용하여 개별적으로 연구의 목적 및 방법, 개인정보의 비밀 보장, 자발적인 연구 참여의 동의 및 거부 권리를 설명하였고, 연구 참여 자체가 학업 및 평가와 무관함을 설명하였다. 연구에 대한 충분한 설명을 들은 후 연구의 목적과 내용을 이해하고 연구 참여에 동의한다는 서면 동의서를 작성한 후, 설문조사를 진행하였다. 또한 개인정보 보호를 위해 코드로 대상자의 정보를 식별하였다. 연구자는 연구와 관련된 대상자의 개인적 정보와 조사 자료는 오직 연구 목적으로만 사용할 것임을 설명하였다. 설문지 작성에 소요되는 시간은 5–10분 정도였고, 완성한 설문지는 바로 회수용 봉투에 넣은 후 밀봉된 상태로 회수하였다. 연구에 참여한 대상자에게 감사의 표시로 소정의 선물을 제공하였다. 필요한 경우 정서적 지지와 상담 가능한 핫라인, 교내 상담센터와 연계할 수 있도록 안내하였다.

### 자료 분석

수집된 자료는 IBM SPSS Statistics for Windows ver. 23.0 (IBM Corp., Armonk, NY, USA) 프로그램을 이용하였다.

1) 대상자의 일반적 특성과 월경 관련 특성은 기술 통계를 이용하여 분석하였다.

2) 대상자의 우울, 피로, 신체존중감, 월경 전 증상 정도는 평균과 표준편차로 분석하였다.

3) 대상자의 일반적 특성, 월경 관련 특성 및 우울 정도에 따른 월경 전 증상의 차이는 독립표본 t-test, ANOVA로 분석하였다.

4) 대상자의 우울, 피로, 신체존중감과 월경 전 증상의 상관관계는 Pearson correlation coefficient로 분석하였다.

5) 대상자의 월경 전 증상에 영향을 미치는 요인은 다중 회귀분석(multiple re¬gression analysis)으로 분석하였다.

## Results

### 대상자의 우울, 피로, 신체존중감, 월경 전 증상 정도

대상자의 평균 우울 점수는 16.05±7.72점이었으며, 우울 점수에 따른 분류에서 경미한 우울(16–20점)은 24명(16.6%), 중한 우울(21–24점)은 22명(15.2%), 심한 우울(25점 이상)은 14명(9.7%)으로 나타났다. 피로는 문항 평균 4.84±0.84점으로 중등도 수준이었으며, 신체존중감도 문항 평균 2.94±0.44점으로 중등도 수준이었다. 하위 요인에서 신체기능존중감 3.22±0.59점, 외모존중감 2.59±0.58점, 체중존중감 2.81±0.83점으로 나타났다. 월경 전 증상의 문항 평균은 2.52±0.92점으로 낮은 수준이었고, 하위 영역별로는 통증이 2.95±1.14점으로 가장 높았으며, 행동 변화 2.92±1.18점, 수분 축적 2.70±0.90점, 부정 정서 2.66±1.27점, 자율신경 반응 변화 2.11±0.83점, 집중력 관련 증상 2.05±1.10점 순으로 나타났다(Table 1).

### 대상자의 일반적 특성, 월경 관련 특성 및 우울 정도에 따른 월경 전 증상 정도 차이

대상자의 일반적 특성과 월경 관련 특성 및 우울 정도에 따른 월경 전 증상 정도 차이를 검증한 결과 월경 주기가 불규칙한 경우(t=2.46, *p*=.015)와 월경통을 호소하는 경우(t=2.74, *p*=.007)에 월경전증후군 점수가 유의미하게 높은 것으로 나타났다(Table 2).

### 대상자의 우울, 피로, 신체존중감, 월경 전 증상의 상관관계

대상자의 월경 전 증상은 우울(r=.58, *p*<.001)과 중간 강도의 유의미한 양의 상관관계가 있는 것으로 나타났다. 또한 피로(r=.33, *p*<.001)는 약한 강도의 유의미한 양의 상관관계가 있었으며, 신체존중감(r=–.28, *p*<.001)과 약하게 유의미한 음의 상관관계가 있는 것으로 나타났다. 또한 우울은 피로(r=.34 *p*<.001)와 양의 상관관계를 보였고, 신체존중감(r=–.24, *p*=.003)과 음의 상관관계가 있었으며, 피로는 신체존중감(r=–.25, *p*=.002)과 음의 상관관계가 있는 것으로 나타났다(Table 3).

### 대상자의 월경 전 증상에 영향을 미치는 요인

대상자의 월경 전 증상에 영향을 미치는 요인을 파악하기 위해 회귀분석을 실시하기 전 다중 공선성 확인 결과 공차한계가 .81에서 .94로 0.1값보다 컸으며, 분산팽창인자(variance inflation factor)는 1.06–1.23 범위로 10 이상을 넘지 않아 다중 공선성 문제를 배제할 수 있었다. 또한 잔차의 독립성 검정을 위해 Durbin-Watson 값을 구한 결과 1.93로 자기상관의 문제는 없었다. 우울, 피로, 신체존중감과 대상자의 월경전증후군에 유의한 차이를 보였던 월경 주기의 규칙성 여부와 월경통 유무는 가변수 처리하여 다중 회귀분석을 실시한 결과 우울(β=.47, *p*<.001), 월경통(β=–.18, *p*=.009), 월경 주기(β=.17, *p*=.013), 신체존중감(β=–.14, *p*=.038)이 통계적으로 유의미한 영향을 미치는 변인으로 나타났다. 회귀모형은 통계적으로 유의하였으며(F=19.83, *p*<.001), 수정된 결정계수(adjusted R2)로 살펴본 모형의 설명력은 41.0%로 확인되었다(Table 4).

## Discussion

본 연구는 간호대학생의 우울, 피로, 신체존중감 정도를 파악하고, 이들 변수가 월경 전 증상에 미치는 영향을 확인하여 간호대학생의 월경 전 증상 관리를 위한 기초자료를 마련하고자 시도되었다.

본 연구 결과 간호대학생의 월경 전 증상 정도는 2.52±0.92점(범위, 1–6점)으로 나타났다. 동일한 도구를 사용하여 중·고등학생을 대상으로 시행한 연구에서[27] 월경 전 증상은 2.03±0.79점으로 본 연구 결과보다 낮은 수준을 보였다. 이를 비교하면 여대생의 경우 중·고등학생보다 월경 전 증상 정도가 높은 것으로 나타났으며, 연구 도구는 다르지만 20대 여성의 경우 여고생에 비해 월경 전 증상이 높게 나타난 Kim 등[4]의 연구 결과를 지지하였다. 하위 영역별로 살펴보면 본 연구에서는 통증이 가장 높은 점수를 보였으며, 그 다음으로 행동 변화, 수분 관련 증상 순이었다. 연구 도구는 다르지만 간호대학생을 대상으로 한 선행연구[28]에 따르면 수분 정체가 가장 높은 점수를 보였으며, 동일한 도구를 사용하여 여대생을 대상으로 한 연구에서도[18] 수분 정체가 높은 점수를 보여 정서적 증상에 비해 신체적 증상과 관련한 불편감이 높음을 알 수 있었다. 하지만 여대생을 대상으로 시행한 Wang 등[29]의 연구에서는 심리·정서적 증상이 가장 심한 것으로 나타나 월경전증후군은 복합적이고 다양한 증상이 보고되고 있음을 알 수 있다. 국제질병분류(International Classification of Diseases, 10th revision) 진단 기준에 따르면 월경전증후군은 경미한 정신적 증상, 복부 팽만, 체중 증가, 유방 압통, 근육통, 집중력 감소, 식욕 변화 중 적어도 한 개 이상의 증상이 황체기에 국한되어 나타날 때로 정의하고 있으며, 본 연구 결과 모든 대상자들이 한 가지 이상의 증상을 호소하는 것으로 나타났다. 이처럼 여성들은 월경 전 증상으로 인해 많은 불편감을 호소하지만 의료적 도움을 받는 경우는 낮은 수준으로 보고되며[5], 특히 여대생은 여성 생식기 검사와 증상 치료에 소극적이다. 따라서 월경 전 증상을 효과적으로 관리하기 위해 개별적 접근이 용이하고 쉽게 이용 가능한 월경 전 증상 자가기록이 중요하며 월경 주기 동안의 변화추이를 확인함으로써 월경전증후군에 해당된다면 자가관리 및 보조적 조치가 필요하다. 또한 월경 전 증상의 중증도 분류에 따른 중재 가이드라인이 제공되어야 할 것이다.

본 연구 결과 간호대학생의 월경 전 증상에 영향을 미치는 요인은 우울, 월경통, 불규칙한 월경주기, 신체존중감이었으며 그 중 우울은 가장 큰 영향을 미치는 변수로 나타났다. 대학생을 대상으로 우울과 월경 전 증상을 살펴본 연구는 찾기 어려웠지만, 여고생을 대상으로 시행한 연구에서[8] 우울은 월경전증후군과 유의한 관련이 있는 것으로 나타나 본 연구 결과를 지지하였다. 특히 청소년기에 월경전증후군은 사회·심리적 요인의 영향을 많이 받는다고 하였는데[9], 후기 청소년기에 해당하는 대학생들 역시 심리적 요인의 영향을 많이 받고 있음을 본 연구 결과를 통해 확인할 수 있었다. 특히 우울은 간호대학생의 정신건강 문제 중 높게 나타나는 증상이므로[12], 우울에 대한 효과적인 중재는 월경 전 증상 감소에 도움을 줄 수 있을 것으로 생각된다.

또한 월경 관련 특성에 해당하는 월경 주기와 월경통은 대상자의 월경 전 증상에 영향을 미치는 것으로 나타났다. 이는 월경 주기가 불규칙한 경우와 월경통이 있는 경우 월경 전 증상 정도가 높음을 의미한다. 간호대학생을 대상으로 시행한 선행연구에서 월경통은 월경전증후군에 유의한 영향을 미치는 것으로 나타나 본 연구 결과를 지지하였다[28,29]. 본 연구 대상자는 월경통이 있는 경우가 66.9%였으며, 간호대학생을 대상으로 한 Kang [28]의 연구에서는 79.9%, Choi 등[30]의 연구에서는 78.2%가 월경통을 호소하는 것으로 나타나 대다수의 간호대학생이 월경통을 경험하는 것을 알 수 있었다. 또한 월경통 수준이 심각할수록 월경 전 증상 점수가 높은 것으로 보고되었다[29]. 여대생의 월경통 해결 방법은 휴식이나 진통제 복용, 참기, 이완요법 등을 주로 활용하고 있는 것으로 나타났으며[9], 그 중 진통제 복용 방법은 휴식이나 참기, 이완요법을 활용하여 월경통을 해결하는 방법에 비해 월경전증후군 증상 정도가 높은 것으로 나타나[9], 월경통 완화를 위한 보다 적극적인 태도가 필요하다.

간호대학생을 대상으로 한 Choi 등[30]의 연구에서 월경 주기는 월경 전 증상의 유의한 영향요인이 아닌 것으로 나타나 본 연구 결과와 상반된 결과를 보였으나, Wang 등[29]의 연구에서 월경 주기가 불규칙한 경우 월경전증후군 정도가 높은 것으로 나타나 이들 변수의 관계를 살펴볼 필요가 있다. 또한 본 연구 대상자의 월경 주기는 불규칙적인 경우가 42.1%로, Choi 등[30]의 연구에서 보고한 21.8%보다 월경 주기가 불규칙적인 대상자의 비율이 높아 이에 따른 차이가 발생하였으리라 생각되며 추후 반복 연구를 통해 월경 주기와 월경 전 증상과의 관계 확인이 필요할 것으로 생각된다.

마지막으로 신체존중감은 월경 전 증상에 영향을 미치는 요인이었으며, 신체존중감이 낮을수록 월경 전 증상 정도가 높은 것으로 나타났다. 관련 선행연구가 없어 직접적인 비교가 어렵지만 신체존중감은 인간의 몸에 대한 광범위한 행동영역으로 주관적 안녕감에 큰 영향을 미치기 때문에[14], 자신의 신체를 존중하는 경우 월경 전 증상들을 편안하게 긍정적으로 받아들일 수 있을 것이다. 반면, 자신의 신체에 대해 불만족하고 부정적으로 평가할수록 스트레스 수준이 높아지게 되며[15], 이러한 스트레스는 월경 전 증상을 더 악화시킬 수 있다[6]. 특히 20대 초반 여대생에서의 신체존중감은 신체의 기능보다는 외모나 체중에 중심을 두고 있기 때문에[14], 자신의 외모나 체중에 왜곡된 생각을 가질수록 월경 전 증상 정도가 더 심해질 수 있음을 유추할 수 있겠다. 따라서 여대생의 월경 전 증상 정도를 감소시키기 위하여 자신의 신체에 대한 부정적 인식과 평가를 개선시켜 신체존중감을 향상시킬 필요가 있다.

이상의 결과를 통해 간호대학생의 월경 전 증상에 영향을 미치는 요인은 우울, 불규칙한 월경주기, 월경통, 신체존중감으로 확인되었다. 따라서 월경 전 증상의 감소를 위해서는 이들 변수를 고려한 다양한 중재 프로그램 개발 및 적용을 통해 월경 전 증상에 대한 적극적인 관리가 필요할 것으로 생각된다.

본 연구는 한 지역에 국한된 대상자를 표본으로 선정하여 표본 편중이 발생할 수 있으며, 월경 전 증상 정도가 비교적 심하지 않은 대상자도 다수 포함되어 있다는 제한점이 있다. 또한 월경통의 강도를 구체적으로 살펴보지 못한 제한점이 있다. 그러나 본 연구에서는 타 집단에 비해 우울과 피로감 호소가 높은 간호대학생을 대상으로 월경 전 증상에 영향을 미치는 요인을 살펴보았으며, 특히 신체존중감이 낮은 시기에 해당하는 대학생을 대상으로 신체존중감과 월경 전 증상의 관련성을 파악하였다는 점에서 선행연구와의 차별성을 갖는다. 본 연구결과를 바탕으로 다음과 같이 제언하고자 한다. 첫째, 다양한 지역 및 학과의 여대생을 대상으로 하는 반복적인 후속 연구가 필요하다. 둘째, 추후 연구에는 월경 전 증상이 심한 대상자를 선정하여 월경 전 증상의 영향요인을 확인해 볼 것을 제언한다. 마지막으로, 본 연구 결과를 바탕으로 간호대학생의 월경 전 증상의 효과적 관리를 위해 우울 관리, 규칙적인 월경 주기, 월경통 완화, 신체존중감 증진을 위한 다양한 프로그램 개발과 그 효과를 규명하는 후속 연구가 필요하다.

## Figures and Tables

**Table 1.** Depression, fatigue, body esteem, and premenstrual symptoms (N=145)

Variable	Categories	Mean±SD	Possible range	Item mean±SD	Item range
Depression		16.05±7.72	0–60	0.80±0.39	0–3
	Normal	85 (58.6)	0–15	0.55±0.12	
	Mild depression	24 (16.6)	16–20	0.90±0.75	
	Moderate depression	22 (15.2)	21–24	1.13±0.06	
	Severe depression	14 (9.7)	25–60	1.65±0.34	
Fatigue		130.63±22.68	27–189	4.84±0.84	1–7
Body esteem		52.99±7.91	18–72	2.94±0.44	1–4
	Function esteem	15.58±3.49	9–36	3.22±0.59	
	Appearance esteem	12.72±2.74	6–24	2.59±0.58	
	Weight esteem	8.42±2.50	3–12	2.81±0.83	
Premenstrual symptoms		94.12±34.70	37–222	2.52±0.92	1–6
	Changes of the autonomic nervous system	12.66±4.99	6–36	2.11±0.83	
	Pain	17.71±6.88	6–36	2.95±1.14	
	Changes of behavior	14.69±5.96	5–30	2.92±1.18	
	Concentration	16.39±8.83	8–48	2.05±1.10	
	Negative emotions	21.31±10.15	8–28	2.66±1.27	
	Water retention	10.80±3.62	4–24	2.70±0.90	

**Table 2.** Differences in premenstrual symptoms according to general and menstrual characteristics (N=145)

Variable	Categories		n (%)	Mean±SD	t or F (*p*)
Age (year)	≤20		83 (57.3)	2.53±0.98	2.86 (.061)
	21–22		46 (31.7)	2.39±0.81	
	≥23		16 (11.0)	3.04±0.98	
		Mean±SD	20.66±1.90		
Sleep time (hour)	≤5		24 (16.6)	2.66±0.97	1.68 (.191)
	6–7		92 (63.4)	2.44±0.85	
	≥8		29 (20.0)	2.78±1.13	
		Mean±SD	6.74±1.36		
Drinking (time/month)	None		30 (20.7)	2.68±1.00	1.89 (.155)
	1–2		95 (65.5)	2.44±0.89	
	≥3		20 (13.8)	2.83±0.98	
Body mass index (kg/m^2^)	<18.5		25 (17.2)	2.79±1.01	1.63 (.199)
	18.5–22.9		93 (64.1)	2.42±0.82	
	≥23		27 (18.6)	2.49±1.05	
Age of menarche (year)	≤12		69 (47.6)	2.63±0.92	1.02 (.364)
	13–14		58 (40.0)	2.52±0.99	
	≥15		18 (12.4)	2.28±0.80	
		Mean±SD	12.75±1.30		
Menstrual cycle	Regular		84 (57.9)	2.38±0.32	2.46 (.015)
	Irregular		61 (42.1)	2.77±1.04	
Menstrual period (day)	3–4		30 (20.7)	2.32±0.99	1.06 (.348)
	5–6		105 (72.4)	2.60±0.94	
	≥7		10 (6.9)	2.61±0.94	
Amount of menstruation	Little		17 (11.7)	2.66±1.10	2.99 (.053)
	Moderate		103 (71.0)	2.43±0.84	
	Much		25 (17.2)	2.92±1.09	
Dysmenorrhea	Yes		97 (66.9)	2.69±0.92	2.74 (.007)
	No		48 (33.1)	2.25±0.89	
Family history of premenstrual syndrome	Yes		88 (60.7)	2.62±0.95	1.19 (.249)
	No		57 (39.3)	2.43±0.91	

**Table 3.** Correlations among depression, fatigue, body esteem, and premenstrual symptoms (N=145)

Variable	r (*p*)		
	Depression	Fatigue	Body esteem
Fatigue	.34 (<.001)		
Body esteem	–.24 (.003)	–.25 (.002)	
Premenstrual symptoms	.58 (<.001)	.33 (<.001)	–.28 (<.001)

**Table 4.** Factors influencing premenstrual symptoms (N=145)

Variable	B	SE	β	t	*p*
(Constant)	2	0.63		3.13	.002
Menstrual cycle regularity^†^	0.3	0.12	.17	2.5	.013
Dysmenorrhea^†^	–0.34	0.13	–.18	–2.63	.009
Depression	0.55	0	.47	6.61	<.001
Fatigue	0.04	0	.09	1.31	.190
Body esteem	–0.16	0	–.14	–2.10	.038
Adjusted R^2^=.41, F=19.83, *p*<.001					

† Dummy variable references were menstrual cycle (irregular) and dysmenorrhea (no).

## References

[1] The American College of Obstetricians and Gynecologists (2015). Premenstrual syndrome [Internet]. https://www.acog.org/patient-resources/faqs/gynecologic-problems/premenstrual-syndrome.

[2] Direkvand-Moghadam A, Sayehmiri K, Delpisheh A, Kaikhavandi S (2014). Epidemiology of premenstrual syndrome (PMS)-a systematic review and meta-analysis study. J Clin Diagn Res.

[3] Lim H, Park Y (2010). Differences in dietary intake and life-style of female college students in seoul with and without premenstrual syndrome. J Menopausal Med.

[4] Kim MJ, Nam YS, Oh KS, Lee CM (2003). Some important factors associated with premenstrual syndrome influence of exercise, menarche, and age on PMS. Korean J Growth Dev.

[5] Lee SR, Lim JY, Lee EJ, Jo YT, Ra CR (2014). Preconception care and policy recommendations for unmarried women of childbearing age: focusing on medical service use and online reproductive health information.

[6] Jang HJ, Sung MH (2018). Impact of menstrual attitudes, premenstrual syndrome, and stress response on quality of life among nursing students. Korean J Women Health Nurs.

[7] Rapkin AJ, Akopians AL (2012). Pathophysiology of premenstrual syndrome and premenstrual dysphoric disorder. Menopause Int.

[8] Lee JY, Kim SM, Kang SH, Chung HG, Choi JH, So HS et al (2016). Association of premenstrual syndrome and premenstrual dysphoric disorder with depression, sleep quality and sleep pattern in the Korean female high-school students. Anxiety Mood.

[9] Jung GS, Oh HM, Choi IR (2014). The influential factors on premenstrual syndrome college female. J Korea Acad Ind Coop Soc.

[10] Park BY, Shim OS (2016). Predictors of quality of life in nursing students and general college students. J Learn Cent Curric Instr.

[11] Kim KH, Yoon HS (2013). Factors influencing sleep quality in nursing students and non nursing students. J Korean Acad Psychiatr Ment Health Nurs.

[12] Park MH, Hwang EH (2016). Comparison of sleep quality, fatigue and depression among nursing students between school lessons and clinical practicum term. J Korean Soc Sch Community Health Edu.

[13] Park SJ, Choi JY (2016). Factors affecting clinical practice-related fatigue among nursing students. J Korea Content Assoc.

[14] Gim WS (2007). Korean overall body esteem scale(KOBES): development, validation, and gender differences. Korean J Psychol Woman.

[15] Hong M, Park Y, Chen EY, Yun JW, Oh MH (2017). Body esteem, stress, and health promoting behavior among korean adults in a community. Korean J Rehabil Nurs.

[16] Ussher JM, Perz J (2020). “I feel fat and ugly and hate myself": self-objectification through negative constructions of premenstrual embodiment. Fem Psychol.

[17] Lee SJ, Lee EJ, An K (2019). A relationship on body esteem, self esteem and eating behavior of nursing students. Asia-pacific J Multimed Serv Convert Art Humanity Social.

[18] Yoon JW (2019). Effects of perceived stress, ego-resilience on premenstrual syndrome in female college students. J Korean Content Assoc.

[19] Moos RH (1968). The development of a menstrual distress questionnaire. Psychosom Med.

[20] Kim HW, Kwon MK (2004). Study on the validity and reliability of MMDQ (Moos Menstrual Distress Questionnaire) among middle school students. Korean Parent Child Health J.

[21] Radloff LS (1977). The CES-D scale: a self-report depression scale for research in the general population. Appl Psychol Meas.

[22] Cho M, Kim K (1993). Diagnostic validity of the CES-D(Korean version) in the assessment of DSM-III-R major depression. J Korean Neuropsychiatr Assoc.

[23] Chon KK, Choi SC, Yang BC (2001). Integrated adaptation of CES-D in Korea. Korean J Psychol Health.

[24] Schwartz JE, Jandorf L, Krupp LB (1993). The measurement of fatigue: A new instrument. J Psychosom Res.

[25] Byeon YS, Lee JI (2004). Reliability and validity tests for the Fatigue Assessment Instrument (FAI). J Korean Acad Fund Nurs.

[26] Gim WS, Cha JH (2006). Body-related values and consumer behavior: an exploratory study for scale development. Korean J Consumer Advert Psychol.

[27] Jeon JH, Hwang SK (2014). A structural equation modeling on premenstrual syndrome in adolescent girls. J Korean Acad Nurs.

[28] Kang DH (2017). Influencing factors in premenstrual syndrome(PMS) among nursing students. J Digit Converg.

[29] Wang HJ, Kang MS, Oh SM (2018). Influential factors on premenstrual syndrome in female college students. Korean Parent Child Health J.

[30] Choi HJ, Kim BM, Kim JW, Kim JH, Oh HJ, Lee GE (2018). Factors associated with stress and menstrual attitude with premenstrual syndrome among nursing students. J Kyungpook Nurs Sci.

